# A Modified Sample Preparation Protocol for High-Efficiency Lab-on-a-Disk-Based Separation and Single-Image Quantification of Soil-Transmitted Helminth Parasite Eggs in Stool

**DOI:** 10.3390/mi16080847

**Published:** 2025-07-24

**Authors:** Mina Wahba, Heaven D. Chitemo, Vyacheslav R. Misko, Doris Kinabo, Matthieu Briet, Jo Vicca, Bruno Levecke, Humphrey D. Mazigo, Wim De Malsche

**Affiliations:** 1 µFlow Group, Department of Bioengineering Sciences, Department of Chemical Engineering, Vrije Universiteit Brussel, 1050 Brussels, Belgium; mina.hany.ibrahim.wahba@vub.be (M.W.); heaven.david.chitemo@vub.be (H.D.C.); doris.kinabo@vub.be (D.K.); matthieu.briet@vub.be (M.B.); 2 SALTO Agro-Bio Research Group, Odisee, University of Applied Sciences, 9100 Sint-Niklaas, Belgium; jo.vicca@odisee.be; 3 Department of Translational Physiology, Infectiology and Public Health, Ghent University, 9000 Merelbeke, Belgium; bruno.levecke@ugent.be; 4 Department of Medical Parasitology, School of Medicine, Catholic University of Health and Allied Sciences, Mwanza 33000, Tanzania; humphreymazigo@bugando.ac.tz

**Keywords:** soil-transmitted helminths, lab-on-a-disk technology, single-image parasite quantification

## Abstract

Soil-transmitted helminths (STHs) present a significant global health challenge, particularly in tropical and subtropical regions. The current diagnostic standard involves the microscopic examination of a stool smear but it lacks sensitivity to detect infections of low intensity. Innovative solutions like lab-on-a-disk (LoD) technologies are emerging, showing promise in detecting low-intensity infections. Field tests conducted using our SIMPAQ (single-image parasite quantification) LoD device have demonstrated its potential as a diagnostic tool, especially for such low-intensity infections. Nevertheless, the device’s efficiency has been limited by significant egg loss during sample preparation, low capture efficiency of eggs within the Field of View (FOV), and the presence of larger fecal debris that obstructs effective egg trapping and imaging. In this study, we conducted a set of laboratory experiments using model polystyrene particles and purified STH eggs to improve the sample preparation protocol. These experiments include the entire SIMPAQ procedure starting from sample preparation, infusing it into the LoD device, centrifugation, delivering the (model) eggs to the FOV, capturing an image, and analyzing it. We analyzed egg losses at each step of the procedure following the “standard” protocol, then elaborated and tested alternative, more efficient procedures. The resulting modified protocol significantly minimized particle and egg loss and reduced the amount of debris in the disk, thus enabling effective egg capture and clear images in the FOV, increasing the reliability of the diagnostic results.

## 1. Introduction

Soil-transmitted helminths (STHs) are a group of intestinal parasitic worms that infect humans through contaminated soil containing eggs from human feces. The most important STHs include the giant roundworm (Ascaris lumbricoides), the whipworm (Trichuris trichiura), and hookworms (Ancylostoma duodenale and Necator americanus), affecting approximately 24% of the world’s population (~1.5 billion people), particularly in the subtropical and tropical regions of Africa, South Asia, and South America [1]. The incidence of these diseases is closely linked to poor socio-economic conditions, such as inadequate access to clean water and sanitation, poor hygiene, overcrowding, and insufficient waste management, as well as environmental and climatic factors (e.g., temperature, humidity, and soil variables) that favor the survival of the worm eggs and larvae in the soil [1,2]. The most vulnerable groups to STH infections are women of childbearing age, preschool children, and school-aged children [2,3]. The diseases cause significant morbidity to the community, ranging from physical and cognitive growth impairment to poor pregnancy outcomes, anemia, and surgical complications such as intestinal obstruction [2]. The WHO-recommended control strategy of these infections includes improving water availability, safety, and hygiene. Large-scale deworming through mass drug administration is another equally significant public health intervention for vulnerable groups [4].

The lifecycle of STHs begins with sexual reproduction and egg laying by adult worms in the host’s small intestine for roundworms and hookworms, or the colon for whipworms. Adult worms lay thousands of eggs per day that are released with stool. When stool contaminated with these parasite eggs is deposited on the soil, the eggs of Ascaris and Trichuris can remain viable for months under favorable conditions and subsequently enter the human host through the consumption of egg-contaminated food or water [5]. In contrast, hookworm eggs hatch in soil into larvae that remain viable for weeks and can enter the human host through direct skin penetration, where they migrate to desirable sites and mature into adults that lay eggs [1,5].

The microscopic demonstration of parasite eggs in stool is the primary method for detecting STH infections. Various methods have been developed to demonstrate eggs on stool, including wet mount preparation, Kato–Katz thick smear, and centrifugation-based techniques like the formal ether/ethyl acetate concentration technique. Newer commercialized innovations include flotation-based methods such as the McMaster technique and the FLOTAC/Mini-FLOTAC method [6,7]. Additionally, other diagnostic techniques include serology-based, molecular-based, and culture-based methods [7]. Today, the Kato–Katz thick smear is the diagnostic standard recommended by the WHO for diagnosing STH infections. This technique involves sieving stool samples, placing a small portion on glass slides using a template, and examining it under a microscope. While this method uses a minimal amount of stool, it has low sensitivity, especially for low-intensity infections, unless multiple samples or smears per slide are analyzed [7,8,9].

Advancements in STH control programs are expected to lead to more people experiencing *low-* and *moderate-intensity infections* [4,9]. These typically asymptomatic infections can serve as reservoirs for disease spread if not promptly detected and treated [8]. This underscores the need for *highly sensitive diagnostic methods* to identify these cases. In this regard, the Single Imaging Parasite Quantification (SIMPAQ) device has been designed and developed [10], which employs a lab-on-a-disk (LoD) technology [11,12,13,14,15,16,17,18,19,20,21,22,23], a subclass of integrated Lab-on-a-Chip (LOC) technology [11,16,17,22]. In addition to portability, the use of small amounts of materials and reagents, faster reaction times, and programmability featured by a LOC [16], the LoD technology employs pseudo-forces generated during the rotation of the device: centrifugal force, the Coriolis force, and the Euler force [17]. This enables a wide range of applications demonstrated on a spinning disk [18] including clinical chemistry, immunoassay, cell analysis [12,21], nucleic acid tests [15], sample-to-answer systems for biomedical point-of-care and global diagnostics [13], liquid handling automation for the life sciences, process analytical techniques and cell line development for biopharma as well as monitoring the environment, infrastructure, industrial processes and agrifood [20,23]. The LoD platform has been applied for detection and molecular analysis of pathogens [14] such as, e.g., *Salmonella*, a major food-borne pathogen [14,19].

The SIMPAQ device [10] concentrates and traps parasite eggs using two-dimensional flotation by combining centrifugation and flotation forces. This occurs by adding a saturated sodium chloride flotation solution to the stool sample, which is slightly denser than parasite eggs, causing the eggs to float while most of the stool particles sediment, thus isolating eggs from debris. Subsequently, centrifugation of the disk directs the eggs toward the center of the disk, where they are packed into a monolayer on a converging imaging zone called the Field of View (FOV), allowing for single-image capture using a digital camera, which facilitates the immediate digitalization of the data [10,24]. It is potentially a portable, point-of-care, reusable device that requires only a small amount of stool sample (1 g) and utilizes inexpensive, readily available materials for testing. The technique has a short time to results and demonstrates a strong positive correlation (0.91) with the commercially available Mini-FLOTAC method, with the ability to detect low-egg-count samples with as low as 30 to 100 eggs per gram of feces [10].

Field tests using animal samples (pigs and dogs) to compare the device’s performance against the McMaster and flotation methods showed that it was able to correctly detect over 93% of positive cases (91.39–95.63% sensitivity against McMaster and 91.00–95.35% sensitivity against the flotation method) and could detect eggs in negative samples using the reference techniques, signifying its promising potential as a diagnostic tool in settings of low and moderate infection intensities [25].

In a field test of the SIMPAQ device using human samples in an STH-endemic area (Northern Tanzania), the device demonstrated high specificity and negative predictive values compared to existing recommended tests: the Kato–Katz thick smear and formal ether concentration tests [9]. However, it exhibited low sensitivity due to the significant *egg loss* that occurs during *sample preparation* steps [9], a phenomenon also observed by Sukas et al. [10]. Nevertheless, the exact points of loss and the degree of egg loss remain unknown. Moreover, when assessing the disk’s capture efficiency, despite significant egg movement towards the disk’s center, most did not reach the imaging zone and remained in various locations within the flow chambers. Around 22% of the 71% of eggs that reached the chip were successfully trapped in the imaging zone, thus necessitating an examination of the whole disk in multiple locations and the taking of multiple pictures to obtain an exact fecal egg count in a sample, thereby increasing the time to results [10].

Factors that hindered the successful entry of eggs into the FOV included (i) the presence of additional inertial forces: Coriolis and Euler forces cause the deflection of the path of the eggs, especially near the center of rotation, where the centrifugal force is small, resulting in the eggs colliding with or sticking to the lateral walls and moving in a zigzag pattern or even backwards instead of moving towards the FOV, thus lowering the capture efficiency [26]. Additionally, (ii) the presence of larger fecal debris that passes through the 200 μm filter membrane in the disk hinders the eggs’ entry to the imaging zone. Attempts to improve the disk’s capture efficiency have been made, including changes in the disk’s design. This has taken the form of reducing the length of the channel from 37 mm in initial designs to 27 mm to minimize the effects of the additional Coriolis and Euler forces. Additionally, surfactant has been added to the flotation solution to reduce the adherence of eggs to the walls of the syringes and disk, as well as different centrifugation speeds being tested to identify the ideal rotation speed that will give the highest yield [24].

Thus, the SIMPAQ method has been further improved since the launch of its original version [10], and its yield has been enhanced. This was achieved by further modification of the design of the LoD (based on detailed analysis of the egg/particle motion in the centrifuge chamber and theoretical analysis) combined with the application of surfactants, as explained above. However, while these important modifications provided a substantial improvement in the operation and egg separation/capture efficiency of the method, the overall performance was still negatively affected due to the undesired loss of eggs at different steps of the procedure, i.e., during sample preparation. The sample preparation steps have always been performed following the elaborated “standard” protocol that was optimized in terms of its efficiency, robustness, and simplicity of operation in field conditions (including the minimization of resources like cleaning water). This standard protocol has never been revised (despite some minor modifications). The need for the revision of the standard protocol is due to the fact that it became the main reason for the limited efficiency of the SIMPAQ method, when other restrictions (i.e., due to the LoD design) have been, to a great extent, overcome.

The goal of this study is to systematically analyze egg losses at each step of the sample preparation procedure as part of the overall operation of the SIMPAQ method and to elaborate more efficient (and still robust and resource-saving) steps, determining a new “improved protocol” (cp. to the “standard protocol”) that would allow one to substantially decrease egg losses and, in this way, enhance the efficiency of the SIMPAQ method. To achieve this goal, we focus in this work on protocol improvement in order to (i) quantify and decrease egg losses that occur during the sample preparation steps, and (ii) improve the capture efficiency of the disks in the Field of View by improving the working protocol of testing the samples. For this purpose, we conducted experiments using polystyrene red particles as models for STH eggs and actual STH eggs preserved in ethanol spiked in goat and human stool samples.

## 2. Materials and Methods

### 2.1. Disk Description and Imaging Set-Up

**Figure 1:**
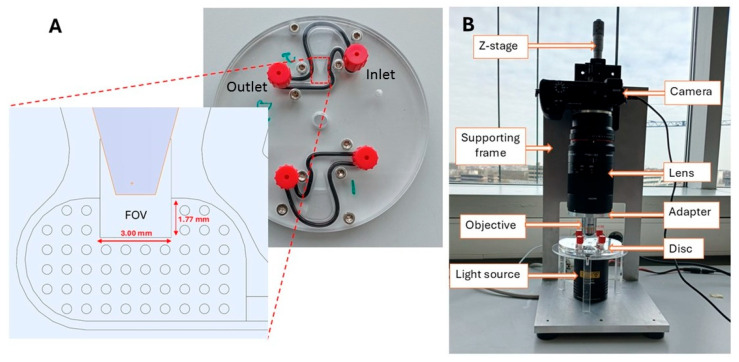
Disk description and imaging set-up. (**A**) The SIMPAQ disk with a zoomed-in computer-aided design showing the Field of View and its dimensions (“large FOV”). Alternative SIMPAQ disk design has an FOV of 1.60 mm × 1.24 mm (“small FOV”). (**B**) Imaging set-up to capture images of helminth eggs in the disk.

The SIMPAQ disk is a circular plastic device made of polymethyl methacrylate (PMMA) with a diameter of 10 cm. It features two diagnostic chambers, each measuring 27 mm in length. These chambers have a sloping depth that decreases from the outer edge towards a rectangular area indicated as the FOV, which is designed to collect and image eggs. The depth of the FOV ranges from 60 to 100 μm and is specifically designed to trap worm eggs (60 and 100 μm) in a monolayer. The dimensions of the FOV are 1.77 mm by 3.00 mm (“large-FOV” disk) or 1.24 mm by 1.60 mm (“small-FOV” disk), which are matched with the image capture settings to enable a single image of the results to be captured (Figure 1A).

The mechanism and background physics of the operation of the LoD device are presented in detail in Refs. [10,24,26]. After the initial preparation procedure, the sample suspended in a flotation solution is infused in the LoD chamber via the inlet (Figure 1A), and the chamber is sealed. The centrifugation for a rotation speed ranging from 800 rpm to 2000 rpm provides an acceleration between 15 *g* (near the FOV) and 32 *g* (near the inlet position) (at 800 rpm) and 94 *g* and 201 *g* (at 2000 rpm) [26]. Here, *g* = 9.81 m/s^2^ is the acceleration due to gravity at the Earth’s surface. Since the egg/particle density (see Section 2.2) is lower than that of the flotation solution (1.175 g/mL), eggs/particles drift towards the FOV, where they are collected, and their image is captured by the camera.

The imaging system features a digital Sony α6100 camera (Sony Group Corporation, Minato, Tokyo, Japan) connected to a Samyang 2.8/100 mm macro lens (Samyang Optics, Masan, Republic of Korea). This macro lens is attached to a 10× magnification objective lens through an adapter. To improve visibility during result analysis, we used a halogen light source (Quartz Tungsten–Halogen lamp, Thorlabs Inc., Newton, NJ, USA) (Figure 1B).

### 2.2. Flotation Solution and Polystyrene Particles

A flotation solution of 1.2 g/mL density was used in these experiments, for which NaCl salt was dissolved in distilled water (Millipore Synergy UV, Spectralab Scientific lnc., Markham, ON, Canada). A 0.05% Tween-20 solution was also added to the flotation solution, serving as a surfactant to reduce the adherence of polystyrene particles and eggs to the apparatus used. Polystyrene (PS) particles (Microparticles GmbH, Berlin, Germany) diluted in distilled water at a 20 particles/µL concentration were used as models for worm eggs in these experiments. The particles had a diameter of 59.8 µm and a density of 1.05 g/mL, which is comparable to helminth eggs: Ascaris eggs (dimensions: 45 to 75 µm (fertilized) and up to 90 µm (unfertilized); density: 1.11 g/mL), hookworm eggs (dimensions: 60–75 µm by 35–40 µm; density: 1.055 g/mL), and Trichuris eggs (dimensions: 50–55 µm by 20–25 µm; density: 1.15 g/mL) [24,27,28,29].

### 2.3. Stool Samples and Purified Helminth Eggs

Both worm-negative goat and human stool samples were used in these experiments. The human stool samples were obtained from Tanzania, whereas the goat stool samples were obtained from Ghent University. To quantify the improvement in the protocol and capture efficiency, known numbers of polystyrene particles (10, 25, 50, and 100) and worm eggs were spiked in the stool samples. These eggs were purified from human stool collected during a worm expulsion study in Pemba Island (Tanzania). For each load, the tests were performed in triplicate.

### 2.4. Stool Sample Analysis Using the Standard Protocol

In the standard SIMPAQ protocol, 1 g of stool sample was mixed with 20 mL of distilled water in a 50 mL Falcon tube and homogenized using plastic granules (Apacor, Berkshire, UK) followed by filtration through stacked polyethylene terephthalate (PET) filters (pluriStrainer^®^ pluriSelect Life Science, Leipzig, Germany) with a 200 µm and 20 µm pore size. The eggs were then rinsed from the 20 µm filter surface using 2 mL of distilled water, collected in a 6-well plate, and then transferred to 2 mL Eppendorf tubes for centrifugation at 1500 rpm for 3 min. The obtained pellet was resuspended in 400 µL of flotation solution and injected into the disks using a 1 mL syringe, where they were centrifuged again at 5000 rpm for 5 min [10].

The capture efficiency of the disks was calculated as the percentage of eggs in the disk that were in the Field of View.

## 3. Results

### 3.1. Protocol Improvement to Enhance Particle Retention in Samples During Preparation

The sensitivity of the SIMPAQ diagnostic disk is reduced due to the significant loss of eggs that occurs during the sample preparation steps [9,10]. To quantify this loss, spiked goat and human stool samples (100 PS particles) were examined using the standard protocol. The materials used in the test—that is, Falcon tubes, filters, six-well plates, and Eppendorf tubes—were analyzed using the imaging set-up to determine the number of particles that remained attached to their surfaces (performed in triplicate for each sample). It was found that, on average, around half of the spiked particles remained in the materials used—57.0% (±11.6% SD (standard deviation)) in case of the goat stool. Therefore, only 43.0% (±7.0%) of the spiked particles reached the disk. For the human samples, 47.3% (±8.2%) of the particles remained in the materials; hence, only 52.7% (±6.8%) of the spiked particles reached the disk. Most of the particles were located in the first filter: 32.0% (±6.6%) for the goat samples and 23.7% (±8.6%) for the human samples (Table 1). This difference in retention can be attributed to variations in stool consistency between goats and humans. Goat stool tends to contain coarser and larger debris, which can more effectively trap polystyrene particles.

**Figure 2:**
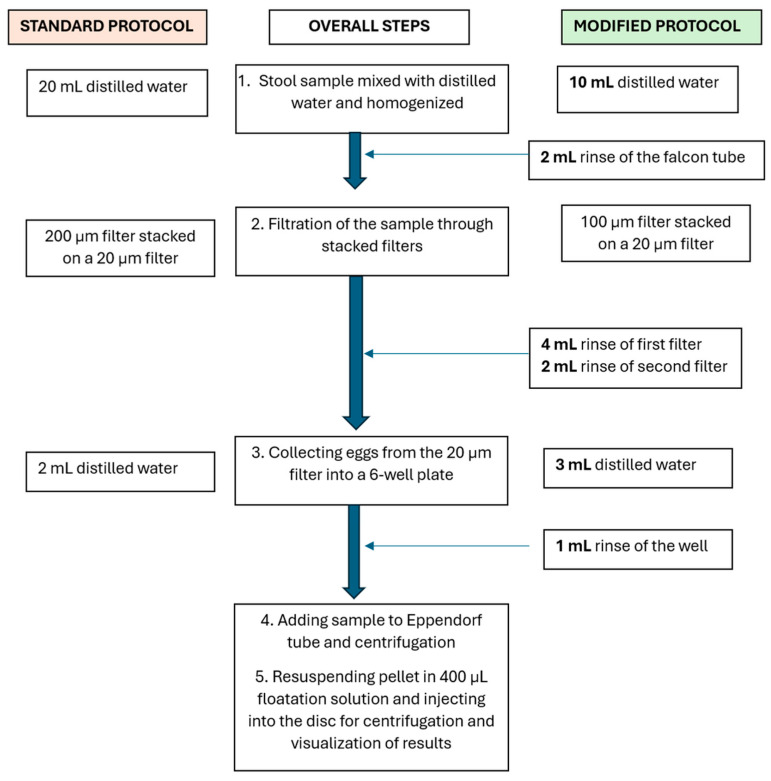
A comparison of the standard and modified sample preparation procedures. In the standard procedure, the stool sample is mixed with 20 mL of water and filtered through 200 µm and 20 µm pore stacked filters. The 20 µm pore filter is washed to collect the trapped eggs, and the sample is centrifuged. The pellet containing the eggs is mixed with 400 µL of flotation solution and injected into the disks. In the modified procedure, the same volume of water is used and distributed in several washing steps.

To address these challenges, the standard protocol was then modified. Instead of mixing the stool samples with 20 mL of distilled water for homogenization, only 10 mL of water was used initially to homogenize the stool sample. The remaining 10 mL was used for washing steps, distributed as follows: 2 mL in the Falcon tube, 4 mL in the first filter, 2 mL in the second filter, and 2 mL in the six-well plate (Figure 2). Additionally, 100 µm filters were utilized as the first filters instead of the 200 µm filters used in the standard protocol to minimize the amount of stool debris that was collected at the second filter and thus introduced in the disk, subsequently interfering with particles and egg movement in the disk.

Each of the stool samples from goats and humans was spiked with 100 PS particles and tested using these modifications. The tests were performed in triplicate. The incorporation of the washing steps improved the number of particles that reached the disk. Specifically, for the goat stool samples, the effectiveness increased from 43.0% (±7.0%) to 71.3% (±4.7%), and for the human stool samples, it rose from 52.7% (±6.8%) to 76.3% (±8.7%) (Table 1). Minimizing egg loss during the sample preparation step is crucial, especially when dealing with low-intensity infections where the number of eggs per gram of feces is low, that is, 1–4999 for Ascaris, 1–999 for Trichura Trichuris, and 1–1999 for hookworms [30].

**Figure 3:**
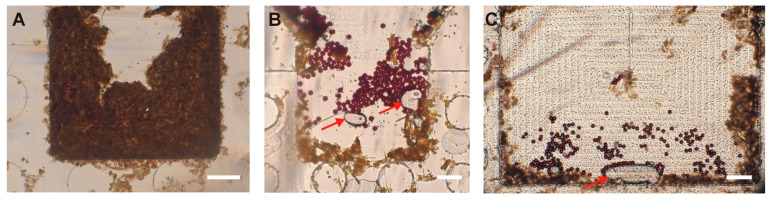
The effect of the initial amount of stool sample on the clarity of images. (**A**) 1 g stool sample. (**B**) 0.5 g stool sample. (**C**) 0.25 g stool sample. Clearer images were obtained with smaller stool samples. The images shown in panels (**A**,**B**) are for small-FOV (1.60 mm × 1.24 mm) disks, and the image in panel (**C**) is for a large-FOV (3.00 mm × 1.77 mm) disk. Scale bar: 300 µm. The arrows indicate occasional bubbles that can be seen in the Field of View.

We also aimed to determine the appropriate sample weight to use in our protocol. We tested three starting sample weights (1 g, 0.5 g, and 0.25 g), each spiked with 100 PS, and observed the number of residual particles in the sample reaching the FOV and the clarity of the image obtained thereafter in the FOV. For each condition, the tests were performed in triplicate. The number of residual particles in the sample that reached the FOV increased when using a smaller weight, with 0.25 g giving the highest mean of 66.7 (±7.4) (Table 2). Similarly, the amount of debris in the FOV decreased when using smaller weights, enabling the obtaining of clearer images, and hence easy quantification (Figure 3). Therefore, the amount of stool to be used was set as 0.25 g. Excess stool debris was observed to cause filter blockage, thus lengthening the filtration time. Additionally, it led to disk blockage and contributed to the poor delivery of eggs to the FOV, leading to poor-quality images of the disks.

### 3.2. Polystyrene (PS) Particle Distribution in the SIMPAQ Disk

**Figure 4:**
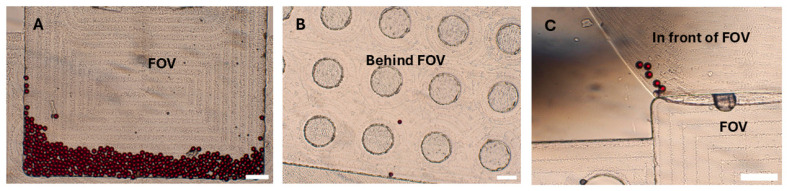
Particle distribution in the disk. An illustration of the potential locations of particles in the disk. (**A**) Polystyrene particles captured in the Field of View (FOV). (**B**) Polystyrene particles behind the Field of View. (**C**) Polystyrene particles in front of the Field of View. Scale bar: 300 µm.

It has been previously reported that not all eggs that reach the disk are captured within the FOV, necessitating the examination of the entire chamber to obtain an accurate count of eggs in a sample [9,10]. We identified three potential locations where eggs or particles may reside within the disk chambers: near the borders of the chamber, the region in front of the FOV, and behind the FOV. To minimize the adherence of eggs and PS particles to the walls of the chamber (both near the borders and in front of the FOV), we used 0.05% Tween-20 as a surfactant. Additionally, the escape of eggs and PS particles behind the FOV can be reduced by selecting disks with appropriate dimensions, specifically, around 20 µm in depth in this region. This allows only smaller debris and air to pass while physically restricting the escape of eggs from the FOV. Despite these considerations, some particles (17.3%) were still occasionally observed in potential locations of the disk, that is, the region in front of the FOV (8.3%), followed by locations near the chamber borders (5.3%) and regions behind the FOV (3.7%) (Table 3; Figure 4). Efforts were made to increase the concentration of Tween-20 surfactant in the flotation solution to further prevent particle adherence to the walls. However, this led to the generation of excessive bubbles during disk centrifugation, which ultimately hindered the effective trapping of eggs within the Field of View. Therefore, the concentration was maintained at 0.05%. Furthermore, the disks were fabricated using computer numerical control (CNC) milling (Datron Neo, Datron, Livermore, CA, USA) from PMMA (Eriks, Antwerpen, Belgium). Achieving precise milling depths, especially very small depths like those required for our disks, is challenging. This difficulty is influenced by (i) material-based factors, such as the type of material and inherent surface irregularities, and (ii) machine-based factors, including machine resolution, accuracy, precision limits, and deflections during operation. All these elements can contribute to a lack of exact 20 µm depths behind the FOV [31]. To mitigate this challenge, one approach used was the milling of the PMMA surfaces to create a uniform reference plane prior to the milling of the chambers, along with confirmatory depth testing after fabrication using a stylus profilometer (DektakXT, Bruker, Kontich, Belgium). However, care must be taken when using these disks to examine the potential locations where eggs may occasionally be found to obtain precise results.

### 3.3. Capture Efficiency of the SIMPAQ Disks in the FOV

**Figure 5:**
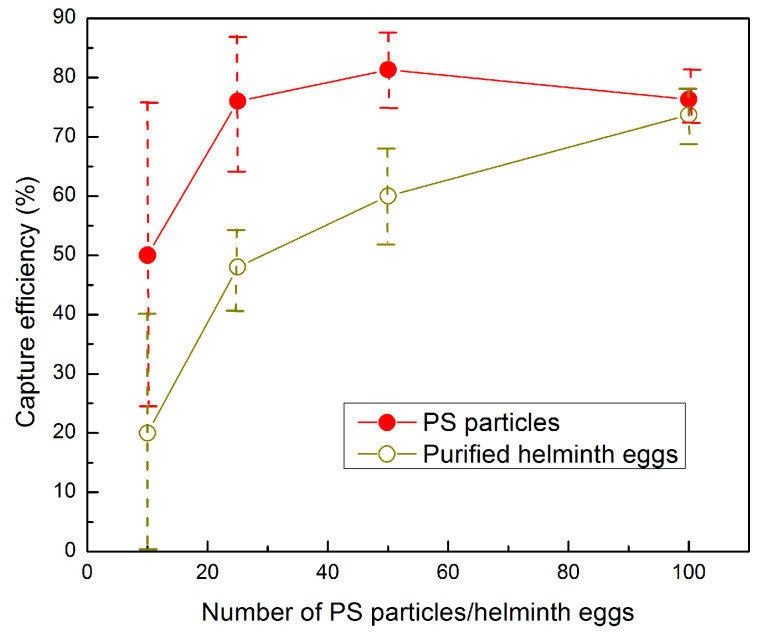
The capture efficiency of the disk FOV versus the number of spiked PS particles or eggs, spiked in worm-negative human stool samples, when using the modified protocol.

**Figure 6:**
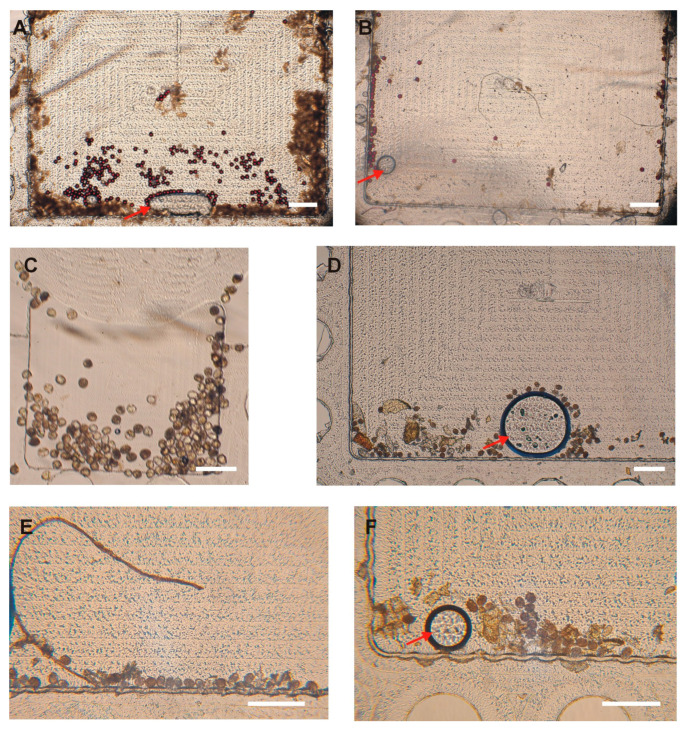
The efficiency of the capture of polystyrene particles (**A**,**B**) and Ascaris and Trichuris eggs (**C**–**F**) spiked in human stool in the Field of View (FOV) using the modified protocol. (**A**) A total of 100 polystyrene particles spiked in human stool. (**B**) A total of 50 polystyrene particles spiked in human stool. (**C**) A total of 100 Ascaris and Trichuris eggs spiked in human stool. (**D**) A total of 50 Ascaris and Trichuris eggs spiked in human stool. (**E**,**F**) A total of 25 Ascaris and Trichuris eggs spiked in human stool. Images shown in panels (**A**,**B**,**D**–**F**) are for a large-FOV (3.00 mm × 1.77 mm) disk, and image (**C**) is for a small-FOV (1.60 mm × 1.24 mm) disk. Scale bar: 300 µm. The arrows indicate occasional bubbles that can be seen in the FOV.

To evaluate the capture efficiency of the disks, that is, the percentage of particles or eggs in the FOV out of the total spiked particles or eggs, we used the modified protocol and the considerations mentioned above. Two sets of experiments were conducted: in the first experiments, varying numbers of PS particles (100, 50, 25, and 10) were spiked in worm-negative human stool samples. In the second set of experiments, stool samples were spiked with similar amounts of purified helminth eggs. The eggs and PS particles were added to the sample, and all preparation steps were carried out according to the modified protocol. Each test was repeated three times. On average, for 100 spiked PS particles, 76.3% (76.3 ± 4.0) were in the FOV. We note that such a small number of eggs (particles in the tests) corresponds to a very low level of infection, and the tests showed a high efficiency of capturing the particles. To go even below that level, in order to examine the lower limit of infection that can be detected by the device (ultimately targeting one egg), we examined the performance of the disk for near-ultra-low levels by spiking 10 particles, where 50.0% (5.0 ± 2.6) of them were in the FOV (Table 4). Similarly, for worm eggs, 73.7% (73.7 ± 4.5) of the 100 spiked eggs were in the FOV. When fewer eggs were spiked in the sample, this efficiency declined to 60.0% (30.0 ± 4.0) for 50 helminth eggs, 48.0% (12.0 ± 1.7) for 25 eggs, and 20% (2.0 ± 2.0) for 10 eggs (Table 4 and Figure 5). The images obtained in the FOV were clear, and both eggs and particles could be easily counted (Figure 6). The remaining eggs were distributed in the disk near the chamber borders, in front of the FOV, and behind the FOV.

This is a significant improvement as compared to the previously recorded efficiency observed by Sukas et al. [10], where around 31% (±25) of eggs in the disk reached the FOV. The successful entry of eggs into the FOV in that earlier study was limited by several factors, including the presence of larger fecal debris in the disk that hindered the eggs’ entry into the imaging zone. This was addressed in the current protocol via the use of a filter with a smaller pore size, i.e., 100 µm instead of the previously used 200 µm. Furthermore, the reduction in the amount of stool samples used in the tests—0.25 g instead of the initial 1 g—helped to reduce the amount of debris reaching the disk.

## 4. Conclusions

The developed LOD device is a promising piece of technology for the detection of low-intensity STH infections. However, its performance was limited due to the loss of eggs during sample preparation, thus preventing them from reaching the FOV. The optimization of the sample preparation protocol proposed in this work was aimed at addressing these limitations. We used both model PS particles and worm eggs to illustrate the improvements during the diagnostic analysis using SIMPAQ disks.

Modifications to the protocol, such as the addition of washing steps, considerably increased the percentage of polystyrene particles that reached the disk FOV, thus minimizing losses. As a result of these modifications, for the goat samples, the percentage of polystyrene particles reaching the FOV after the sample preparation steps rose from 43.0% (±7.0) when using the standard protocol to 71.3% (±4.7), and for the human samples, from 52.7% (±6.8) for the standard protocol to 76.3% (±8.7). The use of finer filters with 100 µm pores instead of 200 µm pores and 0.25 g of stool sample also significantly reduced the amount of stool debris that reached the FOV, enabling users to obtain clear images in the FOV. Additionally, the use of a surfactant proved to be effective in minimizing particle adherence within the collection apparatus, further enhancing the delivery of analytes to the disk FOV. As a result of the above improvements, the capture efficiency of helminth eggs in the FOV significantly increased to 73.7% compared to the previously reported efficiency of 31% (±25). Additionally, the improvements further showcase the promise of the device in detecting even ultra-low egg intensity, as shown by its current ability to detect 20% of 10 spiked helminth eggs.

Reducing sample loss and improving the disk’s capture efficiency enhance the reliability of the diagnostic results. The findings highlight the importance of maintaining strict dimensions for the chamber during disk fabrication. We also stress the need for users to carefully and thoroughly examine the entire chamber to avoid missing any eggs that may be present on the disk. This work establishes a foundation for future field studies in STH-endemic areas to assess the reliability of our device as a powerful alternative diagnostic tool for helminths. In a broader view, our goal is to integrate preparation procedures into the LoD operation cycle.

## Figures and Tables

**Table 1:** The fraction of polystyrene particles lost during sample preparation using the standard vs. modified protocol. Each sample was spiked with 100 polystyrene particles.

	Goat Sample		Human Sample	
	PS (%) (±SD) Standard Protocol	PS (%) (±SD) Modified Protocol	PS (%) (±SD) Standard Protocol	PS (%) (±SD) Modified Protocol
Falcon tube	5.0 (±1.0)	1.3 (±0.6)	3.7 (±1.5)	1.7 (±1.5)
First filter	32.0 (±6.6)	17.3 (±7.8)	23.7 (±8.6)	13.3 (±4.5)
Second filter	6.0 (±1.7)	2.7 (±1.5)	4.7 (±2.5)	2.0 (±1.0)
Six-well plate	8.0 (±2.0)	4.3 (±1.2)	8.7 (±3.8)	3.7 (±1.5)
Eppendorf	6.0 (±1.0)	3.0 (±2.0)	6.7 (±2.1)	3.0 (±1.0)
Total particles lost	57.0 (±11.6)	28.7 (±6.6)	47.3 (±8.2)	23.7 (±4.9)
Particles entering the FOV of the disk	**43.0 (±7.0)**	**71.3 (±4.7)**	**52.7 (±6.8)**	**76.3 (±8.7)**

**Table 2:** The number of polystyrene particles reaching the FOV using different weights of stool samples. Each sample was spiked with 100 polystyrene particles.

Attempts	1 g Stool	0.5 g Stool	0.25 g Stool
1	51	48	61
2	40	67	64
3	38	64	75
Mean (±SD)	43.0 (±7.0)	59.7 (±10.2)	66.7 (±7.4)

**Table 3:** The distribution of polystyrene particles in the disk.

Location in the Disk	Mean ± SD	Percentage (n = 200)
FOV	164.7 ± 13.0	82.3
In front of the FOV	16.7 ± 6.1	8.3
Behind the FOV	7.3 ± 1.2	3.7
Near the borders	10.7 ± 3.1	5.3

**Table 4:** The FOV capture efficiency of the disks using a varying number of particles and eggs.

Mean Number of Spiked Polystyrene Particles	Mean Number of Particles in FOV (±SD)	Capture Efficiency (%)
100	76.3 (±4.0)	76.3
50	40.7 (±3.1)	81.3
25	19.0 (±3.0)	76.0
10	5.0 (±2.6)	50.0
**Mean Number of Spiked Eggs**	**Mean Number of Eggs in FOV** **(±SD)**	**Capture Efficiency** **(%)**
100	73.7 (±4.5)	73.7
50	30.0 (±4.0)	60.0
25	12.0 (±1.7)	48.0
10	2.0 (±2.0)	20.0

## Data Availability

The original contributions presented in this study are included in the article. Further inquiries can be directed to the corresponding author.
